# Chronic Exertional Compartment Syndrome in Athletes: An Overview of the Current Literature

**DOI:** 10.7759/cureus.47797

**Published:** 2023-10-27

**Authors:** Mohammed M Tarabishi, Ahmad Almigdad, Shahd Almonaie, Sebastian Farr, Clemens Mansfield

**Affiliations:** 1 Department of Reconstructive Orthopedic Surgery, King Fahad Medical City, Riyadh, SAU; 2 Department of Pediatric Orthopedic and Adult Foot and Ankle Surgery, Orthopedic Hospital Speising, Vienna, AUT; 3 Department of Orthopedics, Royal Medical Services, Amman, JOR; 4 Department of Orthopedic Surgery, Alfaisal University College of Medicine, Riyadh, SAU; 5 Department of Pediatric Orthopedics, Orthopaedic Hospital Speising, Vienna, AUT

**Keywords:** stress fractures, fascial hernias, medial tibial stress syndrome, cecs, paresthesia, muscle cramps, muscle tightness and weakness, pain, intramuscular compartment pressure measurement, chronic exertional compartment syndrome

## Abstract

Chronic exertional compartment syndrome is an incapacitating condition that primarily affects athletes and individuals with high activity levels.

The exact etiology of the condition is unknown to date, but multiple factors play a role in its occurrence. The clinical presentation includes pain, tightness, muscle weakness, paresthesia, and cramps.

Common tools utilized during the diagnostic approach include intramuscular compartment pressure measurement, advanced imaging to exclude other disorder entities, near-infrared spectrometry, and shear wave elastography, with the clinical diagnosis being the gold standard.

Management includes both conservative and surgical options. Conservative treatment includes gait re-training and botulinum toxin injections. Further, the operative treatment has variable approaches and may be combined with conservative modalities.

This article reviews the literature on chronic exertional compartment syndrome and elucidates future recommendations.

## Introduction and background

Chronic exertional compartment syndrome (CECS) is a condition defined as a state of discommodity in the fascial compartments due to increased intracompartmental pressure (ICP). It occurs in patients with intensive physical activity and can affect both the lower and upper limbs. The classic symptoms of CECS are pain, muscle tightness and weakness, muscle cramps, and paresthesia. Symptoms are alleviated by prompt exercise cessation and rest [1,2]. The CECS diagnosis is, however, quite challenging due to the nebulosity of symptoms. The diagnosis is made (i) clinically and (ii) by ICP measurement pre and post-exercise as the gold standard diagnostic test. Nevertheless, the CECS diagnosis might commonly be delayed and even missed because patients may present with minimal to no symptoms during rest and vague findings during the physical examination. This article reviews the current literature on clinical history, diagnosis, treatment, and future recommendations regarding CECS.

## Review

Prevalence

According to Davis et al., CECS constitutes the second most common cause of exercise-induced lower limb pain, showing a 27% to 33% prevalence after medial tibial stress syndrome [3]. CECS affects young athletes, especially avid runners and soccer players, in the lower limbs while the upper limbs can be affected in weightlifters and motorcyclists.

Based on military studies, Velasco et al. reported an incidence of 1 in 2000 persons per year [4]. In the Nwakibu review, CECS was diagnosed in 0.49 per 1000 of the United States Army [1]. The incidence of CECS in the general population is still unknown to date. Compano et al. and De Brujin et al. revealed the same results with young athletic males, with running, skating activities, and soccer games being the most causative [4,5]. Children with metabolic disorders, especially McArdle’s and glycogen storage diseases, are vulnerable to rhabdomyolysis and CECS as well [6].

Etiology

The exact etiology of CECS is unknown; however, multiple factors might contribute to its occurrence. These factors include micro-trauma or micro-injury, myopathies or diseases that affect the muscles, vascular compromise, fascia compliance, and muscle hypertrophy [4].

Pathophysiology

Due to extensive exertion, intramuscular and intra-compartmental pressures are increased within a closed myofascial compartment or multiple compartments. Consequently, this decreases blood flow and causes muscle ischemia, which is manifested by ischemic pain and neurovascular dysfunction of the affected compartment. Moreover, it is also theorized that with exercise, an increased blood flow to the muscle tissue leads to the expansion of the muscles, which may contribute to the increased intra-compartmental pressures and volume [3,4,7,8]. Other factors may contribute to CECS such as decreased venous return, fascia noncompliance, myopathies, and muscular hypertrophy [8].

In healthy individuals, exercise increases intramuscular pressure, which returns to baseline upon cessation of exercise. However, in patients with CECS, the pressure rise is abnormally high during exercise, and it takes a longer duration to go back to baseline, which leads to ischemia due to interrupted blood flow. Increased muscle volume and fascial tightness might contribute to high intra-compartmental pressure. As a result, pain is elicited due to compression of sensory nerve endings [9].

Although many experiments were performed as histological, vascular, and advanced imaging experiments, none contributed to the pathoanatomy and pathophysiology of the CECS. Some patients were found to have lower capillary density compared to asymptomatic people [10,11].

Clinical presentation

CECS symptoms vary depending on the duration and exercise intensity. The classic symptoms of CECS include pain, muscle tightness, muscle weakness, cramps, and paresthesia. Those symptoms appear during the exercise and are relieved once the activity is ceased. Symptoms usually resolve post-exercise in over 15 minutes but can last for hours. Patients are asymptomatic during rest. In addition, no neurovascular deficits or permanent tissue damage are occurring in the compartment [12].

Ding et al. reported that over time the symptoms may start even earlier during sports participation. Having both the pain along tightness is essential to conclude a diagnosis. Lohrer et al. noted an abrupt escalation in the intensity and the volume of the training, which is called a "training error" [13]. After a certain amount of exertion, the pain sensation may involve one or all of the compartments in the limb. The description of the pain is often as "fullness" or a "crampy” pain, whether it is associated with muscle weakness and paresthesia in the involved compartment or not. Bilateral affection is more likely; this is in contrast to the popliteal artery entrapment syndrome and comparable to stress fractures where running is accompanied by pain and patients cannot run.

The diagnosis of CECS is mainly clinical while imaging is done to rule out other or simultaneous disease entities. The patient usually reports no previous trauma or present injury. A thorough physical examination of prior and post-exertional activity should be undertaken. Examination of the patient in both the static and during gait is necessary. In addition, gait assessment in walking motion and running should be included in the examination. Palpation of lower limbs' compartments after the exertional revealed pain, which is not present at rest. A positive pain finding in the inferior region of the posterior calf should guide the diagnosis toward CECS of the deep posterior compartment. Additionally, pulses should be assessed during rest state and post-physical exercise.

Moreover, the physician should pay attention to facial defects forming herniations of the muscles. Atrophy may be recognized in the leg in prolonged cases [1,14]. In the case of unilateral CCES, the patient may experience pain when palpating the anterior tibialis or the peroneal muscles of the leg. It is also significant to add evaluations to the muscle resistance and examine the lower limb's range of motion before and after the exercises to test provocation [1].

Differential diagnosis

Chandwani and Varacallo mentioned shin splints, tibial stress fractures, nerve entrapment disorders, vascular abnormalities, such as claudication and popliteal artery entrapment syndrome, and tendon disorders, such as inflammation and rupture, to be considered during the diagnostic processes and workups. CECS is commonly misdiagnosed as shin splints due to symptoms overlaps [13,15-17].

Tibial stress fractures at the medial cortex should be suspected and excluded using ultrasound imaging or MRI. Fascial hernias caused by defects are usually visible on the ultrasound, with positive pain findings on palpation and further observation. Tendon pathologies and muscle strains are also considered, and ultrasound/magnetic resonance imaging aids in excluding them. Popliteal artery entrapment syndrome usually leads to pain in the proximal calf after exertion and can be investigated by MR angiography or duplex ultrasound. The ankle-brachial index should be calculated to rule out peripheral vascular diseases.

Nerve entrapment can be excluded by thorough sensory examination and nerve-conducting velocity studies [1]. Common peroneal nerve or tibial nerve entrapment can cause decreased sensations in regions of the foot or the distal part of the lower limb. Lumbar radiculopathy is an essential workup too [4]. Other differential diagnoses include tumors, sickle cell disease, and deep venous thrombosis.

Eventually, the CECS can be a diagnosis of exclusion. There are no uniformly accepted, standardized criteria for the diagnosis of CECS. Instead, the diagnosis is established based on a thorough history-taking and clinical physical examination in addition to ruling out possible diagnoses (Table 1) [4].

**Table 1:** Differential diagnosis of exercise-induced leg pain and diagnostic modalities

Category	Diagnoses	Examination
Bone	Stress fractures	MRI, bone isotope scan
	Tumors	MRI, CT
Myofascial, Tendon	Fascial hernias	MRI, Ultrasound
	Medial tibial stress syndrome	MRI, Ultrasound
	Tendon pathologies	MRI, Ultrasound
	Muscle strains	MRI, Ultrasound
Vascular	Arterial (Popliteal artery entrapment syndrome, peripheral arterial disease)	MR angiography, duplex ultrasound. arteriography
	Venous (deep venous thrombosis, venous insufficiency)	Ultrasound
Local nerve compression	Nerve entrapment	Nerve conducting velocity studies
Referred pain	Lumbar radiculopathy	MRI
	Knee	X-ray, MRI
	Ankle	X-ray, MRI
Hematological	Sickle cell disease	Hemoglobin electrophoresis
Infection	Osteomyelitis	MRI, bone isotope scan

Further diagnostic modalities

In CECS, there is a tendency to decline local tissue oxygen saturation levels after exertion. Thereby, non-invasive near-infra-red spectrometry (NIRS) is used to evaluate the oxygen saturation level changes in muscles at rest and after exertion. Studies have demonstrated the difference in tissue oxygenation between healthy controls and CECS patients, with CECS patients having a more declined oxygen saturation level after exertional activity. Despite all, the NIRS is not often used routinely to diagnose CECS even though it is of high potential to confirm CECS correctly [1,18].

Nevertheless, compared to ICP measurement, the NIRS had similar sensitivity and specificity levels, with both forming gold standard diagnosis methods. The limitation of NIRS is the neglecting of the adipose tissue and skin pigmentation impacts during the NIRS conduction. The adipose tissue is metabolically less active than the muscle tissue, making it less prone to tissue ischemia. Thus, in patients with more body adipose tissue overlying, the NIRs curve along with the absolute StO2 values will be less responsive [19,20]. Measurement of the ICP pre and post-exercise is the only current diagnostic tool for both acute and chronic compartment syndromes. The disadvantage of ICP measurement is that it is an invasive method.

Shear wave elastography is a diagnostic tool that uses ultrasound to obtain the tissue’s elastic and stiffness features; this tissue stiffness found on the elastography is termed “shear wave”. A previous study was done to show that the increase in shear wave modules is associated with increased pressure in the anterior tibialis [4].

Microdialysis, a diagnostic procedure used primarily in intensive care and in vivo, is minimally invasive. A semipermeable membrane was described to continually extract water-soluble compounds easily diffusible from the studied tissue's extracellular space. The collected analytes under ultrasound guidance revealed that the lactate, glutamate, and glycerol levels were high following exertional exercise in the peak phase while the exertion did not affect the glucose concentrations [13,21].

Herring et al. described ultrasound as an objective method that is non-invasive and easy to conduct. Sellei and colleagues have demonstrated that the ultrasonic method is more useful in specific populations, such as pediatric and patients with impaired consciousness, as invasive measures, such as needle manometry is challenging in those populations because it is painful and uncomfortable. Therefore, non-invasive techniques form the best method to approach those patients to objectify the increased pressure in the compartment [22].

**Figure 1:**
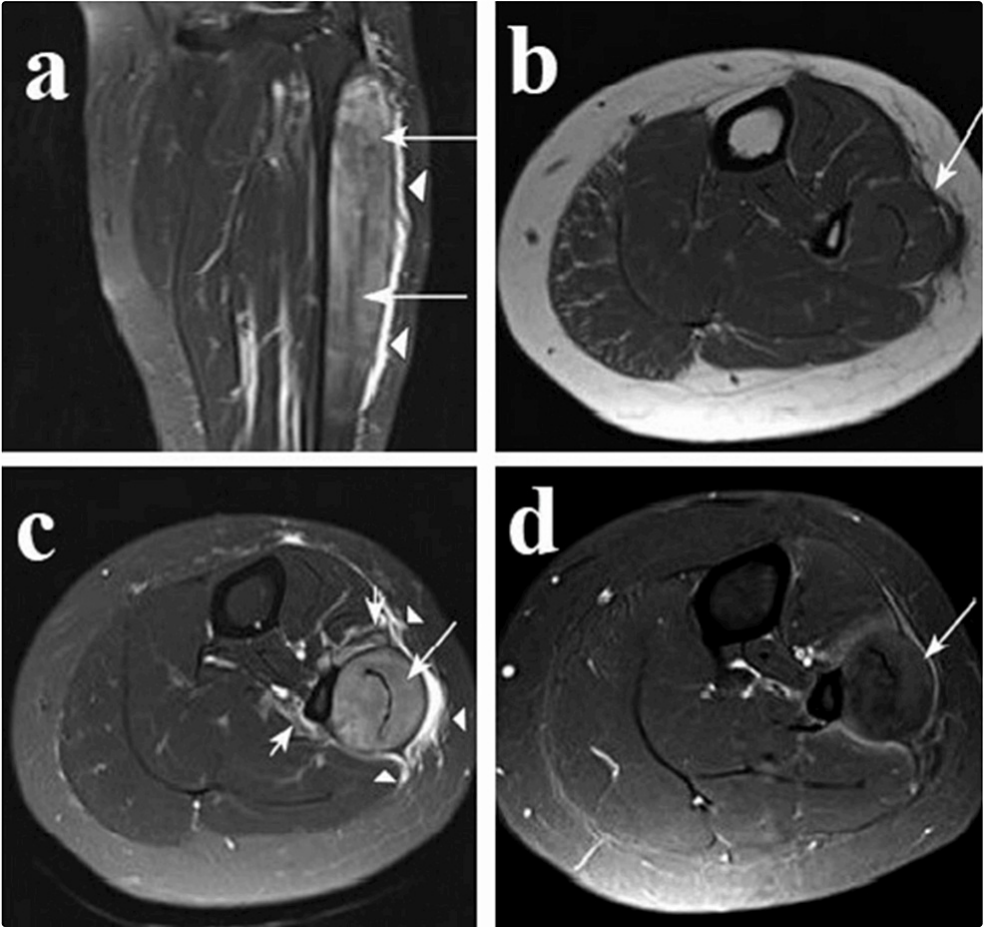
Isolated lateral compartment syndrome of a lower leg (a) Coronal fat-suppressed T2-weighted image shows a diffuse high signal intensity of the peroneus longus and brevis muscles (long arrows). Fluid signal intensity is noted within the surrounding deep and superficial fascial planes (arrowheads). (b) Axial T1-weighted image of the lower leg shows marked swelling of the peroneus longus and brevis muscles with peripheral convex indentation (arrow). (c) Axial fat-suppressed T2-weighted image shows a diffuse high signal intensity of the peroneus longus and brevis muscles (long arrows). Fluid signal intensity is noted within the surrounding deep and superficial fascial planes (arrowheads). Focal increased signal intensity suspected reactive change is noted in the extensor digitorum longus and soleus muscles around the peroneus longus muscle (short arrows). (d) Axial gadolinium-enhanced T1-weighted image shows heterogeneous enhancement within the affected muscles of the lateral compartment (arrow). Reprinted from Creative Commons under (CC BY-NC 3.0), copy and redistributable material [23].

Advanced imaging is important to detect and exclude other pathological changes [5,13]. However, MRI helps differentiate individuals with CECS from individuals without CECS by how water content in the muscles reacts after exertion. In normal situations, the water trapped in the muscle tissue disappears after activity. However, the water stays for a more extended period in patients with CECS. This is usually done by asking the patients to do some activity and motions at the ankle joint level until they develop the symptoms, T2-weighted sequenced images will be captured and obtained of the lower leg. This method has been shown to have a high sensitivity (96%) and specificity (87%) (Figure 1) [4].

MRI is also helpful in detecting periosteal bone marrow edema and differentiating it from end-steal bone marrow edema. MRI is useful for excluding other limb disorders associated with exertion, such as medial tibial stress syndrome (MTSS), fascial defects, stress fractures, DVT, nerve and popliteal artery entrapment, tendinopathy, osteomyelitis, neoplasms, and other anatomical or structural lesions [5,13]. Table 2 highlights the advantages and disadvantages of current diagnostic modalities for CECS.

**Table 2:** Other diagnostic modalities for CECS diagnosis CECS: chronic exertional compartment syndrome

Diagnostic modality	Advantages	Disadvantages
Intracompartmental pressure measurement	Sensitive, ideal	Invasive
Near-infra-red spectrometry	Similar sensitivity and specificity to ICP measurement	Affected by skin pigmentation and amount of adipose tissue
Shear wave elastography	Non-invasive	Non-specific
Microdialysis	Minimally invasive	Non-specific
Compartment fascia flattening pressure	Non-invasive	Difficult in pediatric and uncooperative patients

Treatment

Nonoperative Treatments

The conservative treatment of CECS is offered for three to six months, including massage, physiotherapy, NSAIDs for pain management, and foot orthoses. Foot orthoses are recommended if the patient has foot overpronation (Table 3).

**Table 3:** Treatment modalities for CECS CECS: chronic exertional compartment syndrome

Treatment modality	Treatment option
Conservative	NSAIDS
	Ice
	Activity modification (forefoot running, muscle stretching, warming before exercise)
	Physiotherapy
	Foot orthosis
	Botulinum toxin A
	Extracorporeal shockwave therapy
	Compartment massage
	Fascial fenestration
Operative Treatments	Open fasciotomy
	Endoscopic fasciotomy
	Ultrasound-guided fasciotomy
	Thermal fasciotomy
	Endoluminal release

Botulinum toxin A (BoTN-A) is another option to manage CECS and decreases muscle contraction and secondarily decreases intercompartmental pressure and reduces exertional pain. Teonette et al. reported a case study of a female runner treated conservatively with BoTN-A for anterior and lateral CECS. The pain was alleviated one week after the injection, and the patient was able to jog within two weeks post-injection and remained pain-free after 14 months. Before administering BoTN-A injection, it is recommended to use the lidocaine test, which acts as a map to prevent the administration of BoTN-A in uncertain areas and prevent paralysis [4]. Botox treatment is still controversial although it is a promising method that alleviates exertional pain and decreases pressure. Muscle soreness at the injection site is the initial side effect reported by patients in the first week, and 69% of the patients have reported weakness post-injection.

Important conservative methods include regulating the patient's mobility exercise, but this may be a difficult task to accomplish for professional athletes. Additionally, other nonoperative treatment modalities include extracorporeal shockwave therapy, cryotherapy, muscle stretching, and warming. Nevertheless, nonoperative management will only alleviate symptoms due to muscle rest without curing the main insult [13].

Rajasekaran et al. concluded that compartment massage increases performance and reduces the CECS pain after exercise; this was attributed to fascial stretching and increasing the volume in the selected compartment during the massage [8]. A heel rocker decreases the anterior compartment pressure and changes the gait modality by decreasing the tibialis anterior muscle's eccentric load. Fascial fenestration with ultrasonography was demonstrated as a successful method in treating bilateral anterior and lateral CECS with no known adverse events, in addition to long relief and the ability to participate in sports [8]. Conservatively treated patients reported a lower satisfaction rate (47%) and a low rate of activity return (50%) [24].

Operative Treatments

Fasciotomy can nowadays be considered the gold standard of operative treatment of CECS [1]. The available techniques for fasciotomy include the standard open fasciotomy, endoscopically assisted technique, ultrasound-guided fasciotomy, single incision, and multiple-incision techniques [16,25]. Better surgical outcomes were noted with isolated compartment involvement. On the other hand, lower outcomes are seen in females, the military population, and leg four-compartment fasciotomies. Among the group of patients who underwent the four-compartment fasciotomy, a rate of 64% had a fifth compartment present. Compano et al. mentioned that two-thirds of the young athletes had successful surgical outcomes of CECS treatment, along with 84% of those patients reporting satisfaction in the short- and mid-term. Symptom recurrence after the intervention was rated between 0% and 47%, showing an 8% reoperation rate [4,26]. A four-compartment release was associated with a prolonged duration before fully returning to sports. The pediatric population shows the same outcomes as adults.

Complications that may occur after fasciotomy include hematomas with a rate of 2.7%-22.5%, 2%-18.6% risk of nerve injury, 2.7% risk of deep venous thrombosis, and 0.65%-8.4% risk of symptoms recurrence [12]. Other complications include seroma, saphenous nerve injury, inappropriate fascial release, herniation of the fascia, fascial regrowth, and inability to return to the same fitness level [8].

There are multiple postoperative rehabilitation programs. Miller and Vajapey recommended in the initial 10 postoperative days to accommodate the “PRICE” therapy; protection, rest, ice, compression, and limb elevation to reduce symptoms. In order to establish a successful return to the pre-symptomatic phase before the fasciotomy performance, it is advised to passively stretch, strengthen, neurodynamical mobilization, and biomechanically analyze the respective sport the patient participates in to ensure the same forgoing fitness [6]. Immediate postoperative weight-bearing is advised [7]. Typically, patients return to sports 8-12 weeks postoperatively.

Although endoscopic fasciotomy is a demanding procedure due to the placement of the arthroscope and the instruments in a tight space, it is an efficient technique with minimal healing time, limited morbidities, and better cosmetic outcomes due to the limited number of incisions and decreased recurrence and increased relief [4].

Slimmon et al. noted that 58% of the patients who underwent surgery had lower levels of activity in comparison to the preoperative levels [27]. Waterman et al. [28]. reported 45% of postoperative symptoms recurrence in military personnel [7]. Ding et al. described the fasciotomy as a safe procedure with a 48%-94% satisfaction rate.

Comparison of the outcomes of endoscopic release and the minimally invasive procedure showed mean success rates of 86.3% and 80%, respectively. The success rates associated with the open procedure for anterior CECS and deep posterior CECS are 80% and 30%-65%, respectively. In addition, single-incision approaches were found to show superior outcomes to the 2-incision approach. Open fasciectomy was associated with lower rates of postoperative wound-related complications (6%) and lower rates of recurrence (2%) than subcutaneous fasciotomy wound complications (11%) and recurrence (11%). Overall, no significant variation between the minimal incision open fasciotomy and endoscopic-assisted fasciotomy outcomes has been found.

Two years postoperatively, the anterior compartment CECS success rates were reported to be 81%-100% while it is associated with lower success rates when combined with the concomitant lateral release. Conversely, posterior compartment CECS release was found to have lower success rates ranging between 30% and 65%. Poor outcomes associated with posterior compartment release are unknown, but it is attributed to the natural anatomical complexity and surgical sophistication [8].

The “gold standard” CECS treatment remains decompressive fasciotomy, either in an open or endoscopic manner. Despite that, they have been shown to be associated with high incidences of pain alleviation but an unfortunate risk of recurrence. Other approaches for the same purpose include thermal fasciotomy done with an ablating instrument, associated with a lower rate of complication development and early recovery and attain sports, ultrasound-assisted fasciotomy, transillumination, and endoluminal release [6].

Complications following surgical treatment

The overall reported complication rate is 13%. Ongoing pain, nerve compromise, and vascular complications are reported especially post-multiple compartment fasciotomy. Neurologic dysfunction was most documented as superficial peroneal nerve dysfunction accounting for 5%. Neurologic dysfunction can be due to intraoperative iatrogenic nerve injury or, most commonly, nerve entrapment with scar tissue formation, which is found in 44% of the revision surgery. Infection occurs in 4%, followed by hematoma or seroma (2%), and wound dehiscence occurs at a rate of 1%. Other reported complications were lymphocele, deep vein thrombosis, and nonspecific complications [4].

De Bruijn et al. have noted that the fasciotomy's complication rate can reach 19% [5]. There is a potential difference between civilian and military personnel surgical treatment outcomes with significantly higher recurrence and potential complications in military patients. Therefore, mainly conservative treatment is warranted in military patients [25].

Prognosis

Multiple factors have been studied concerning the prognosis of CECS. One factor is compartment pressures; although it is helpful for diagnosis, there is no strong prognostic correlation to date. Chronicity of symptoms is associated with worse outcomes. According to Davis et al.’s study, the average duration of symptoms before diagnosing the patients was more than two years [3,29]. The factor associated with the best outcomes is the involvement of the anterior compartment. On the other hand, the involvement of the deep posterior compartment was associated with poor results after its release.

Successful and favorable rates can vary between 50% and 100% in civilians and athletes. Worse outcomes were found in military personnel. In this regard, approximately 50% of military personnel who underwent fasciotomy reported recurrence of symptoms, and one out of four patients could not return to the baseline activity prior to CECS treatment. Some patients who underwent anterior release reported residual symptoms three months postoperatively, and some had recurrent pain after an earlier successful fasciotomy [30].

The future of treating CECS

Future prospective studies are highly recommended due to inherent weaknesses associated with retrospective studies in the existing literature. In addition, failure in illuminating previous injuries is associated with the previous retrospective study; thus, future prospective studies are recommended to focus on trauma association as it is related to CECS [3].

Future research should objectify the rehabilitation of CECS cases to maximize the associated outcomes of the operative approach accurately [11]. Future randomized clinical trials studying comparison objectives between nonoperative and operative management would benefit the current CECS medical literature [12].

Moreover, the current evidence on the conservative treatment methods is undoubtedly restricted to case reports and case series; thereby, it is recommended to accommodate higher levels of research study to ascertain the utilization of those methods [8]. In regards to regional anesthesia use, it is advised to conduct more studies in this matter due to the randomized prospective trials controversy of regional anesthesia's safety. Besides, further innovative development of the elasticity measurement with the use of ultrasound should be noted to assess the fasciotomy needs [30].

## Conclusions

The diagnosis of CECS needs high suspicion due to the symptom of nebulosity and its delayed presentation. Measurement of the ICP is the primary diagnostic tool for CECS confirmation. Although fasciotomy is considered the gold standard treatment approach, the recent medical literature noted that fasciotomy's outcomes might not be as fortunate as previously postulated. In summary, an 80% satisfaction rate and return to pre-symptomatic exercise rate in comparison to up to 50% rate associated with conservative management are reported. Military patients demonstrated inferior results after surgery; therefore, many surgeons prefer conservative treatment.
